# Exome sequencing discloses *KALRN* homozygous variant as likely cause of intellectual disability and short stature in a consanguineous pedigree

**DOI:** 10.1186/s40246-016-0082-2

**Published:** 2016-07-16

**Authors:** Periklis Makrythanasis, Michel Guipponi, Federico A. Santoni, Maha Zaki, Mahmoud Y. Issa, Muhammad Ansar, Hanan Hamamy, Stylianos E. Antonarakis

**Affiliations:** Department of Genetic Medicine and Development, University of Geneva, 1 Rue Michel-Servet, 1211 Geneva, Switzerland; Service of Genetic Medicine, University Hospitals of Geneva, Geneva, Switzerland; Department of Clinical Genetics, National Research Centre, Cairo, Egypt; iGE3, Institute of Genetics and Genomics of Geneva, Geneva, Switzerland

**Keywords:** Exome sequencing, Intellectual disability, Short stature, Consanguineous, KALRN

## Abstract

**Background:**

The recent availability of whole-exome sequencing has opened new possibilities for the evaluation of individuals with genetically undiagnosed intellectual disability.

**Results:**

We report two affected siblings, offspring of first-cousin parents, with intellectual disability, hypotonia, short stature, growth hormone deficiency, and delayed bone age. All members of the nuclear family were genotyped, and exome sequencing was performed in one of the affected individuals. We used an in-house algorithm (CATCH v1.1) that combines homozygosity mapping with exome sequencing results and provides a list of candidate variants. One identified novel homozygous missense variant in *KALRN* (NM_003947.4:c.3644C>A: p.(Thr1215Lys)) was predicted to be pathogenic by all pathogenicity prediction software used (SIFT, PolyPhen, Mutation Taster). *KALRN* encodes the protein kalirin, which is a GTP-exchange factor protein with a reported role in cytoskeletal remodeling and dendritic spine formation in neurons. It is known that mice with ablation of *Kalrn* exhibit age-dependent functional deficits and behavioral phenotypes.

**Conclusion:**

Exome sequencing provided initial evidence linking *KALRN* to monogenic intellectual disability in man, and we propose that *KALRN* is the causative gene for the autosomal recessive phenotype in this family.

## Background

Consanguinity is practiced in a large number of human populations with rates reaching 20–50 % in several countries in North Africa and the Middle East [1, 2]. Offspring of consanguineous marriages are at a higher risk of having congenital anomalies caused by pathogenic variants in genes following autosomal recessive inheritance [3–5]. In a study in our laboratory, we employed whole-exome sequencing and genotype analysis to screen members of consanguineous families with likely recessive disorders. Our hypothesis was that because of the homozygosity of the causative defect, the diagnostic strategy would be successful in identifying the molecular basis of the disorder in at least a proportion of the participating patients. Exome sequencing identified the causative variant in up to 34 % of the progeny of consanguineous parents affected by undiagnosed autosomal recessive disorders sequenced in our laboratory [6], as well as a number of novel candidate genes for autosomal recessive disorders [7–9].

Early-onset intellectual disability originating before the age of 18 years with an IQ below 70 is estimated to affect 1–3 % of western populations but could be more common in highly consanguineous populations [10] due to the role of autosomal recessive variants. Autosomal recessive intellectual disability (ARID) is extremely heterogeneous, and causative genes may reach thousands with the vast majority still unknown [10]. With the introduction of NGS technologies, new ID genes are being identified including over 300 genes for ARID [11]. Many of these were identified in studies on consanguineous families. Among 136 consanguineous Iranian families with various forms of ID, pathogenic variants were identified in 78 families including homozygous mutations in 23 genes previously implicated in ID and in 50 candidate genes for ARID [12]. Examples from recent studies on consanguineous families with ID revealing pathogenic variants include variants in TNIK gene [13], SLC6A17 gene [14], and c12orf4 gene [15].

To add to the repository of ARID candidate genes, we report two affected siblings, offspring of a consanguineous marriage, with intellectual disability (ID), hypotonia, short stature, growth hormone deficiency, and delayed bone age. Exome sequencing and homozygosity mapping identified only one strong candidate variant in *KALRN* (NM_003947.4:c.3644C>A: p.(Thr1215Lys)). Kalirin-7, a major isoform of kalirin, is known to regulate spine density in hippocampal and cortical neurons [16], growth hormone functional secretion [17], and bone homeostasis [18].

### Methods

Genotyping, exome sequencing, and variant analysis were performed as previously described [6]. All the family members were genotyped with a dense genotype array in order to define runs of homozygosity (ROH) in all the family members. DNA from the proband was used for exome sequencing, and after calling variants, the ROH were used in order to identify the variants that were in an ROH in the affected siblings, which was not shared by the unaffected sibling and the parents. In more detail, DNA samples from affected family members, their unaffected sibling, and their parents were genotyped using the HumanOmniExpress Bead Chip by Illumina Inc.® (720K SNPs). ROH for every individual were identified using PLINK. We defined as ROH the regions with 50 consecutive SNPs irrespective of the total size of the genomic region, allowing for one mismatch. The ROH region was the one demarcated by the first heterozygous SNPs flanking each established homozygous region. The exome of one affected individual (IV:1) was captured using the SureSelect Human All Exonsv5 reagents by Agilent Inc.®. Sequencing was performed in an Illumina HiSeq 2000 sequencer. Results were analyzed with BWA [19], SAMtools [19], Pindel [20] and ANNOVAR [21], and the exonic variants in combination with the ROH were used by CATCH v1.1 [22] that provided the final list of variants

### Results

#### Clinical report

**Fig. 1:**
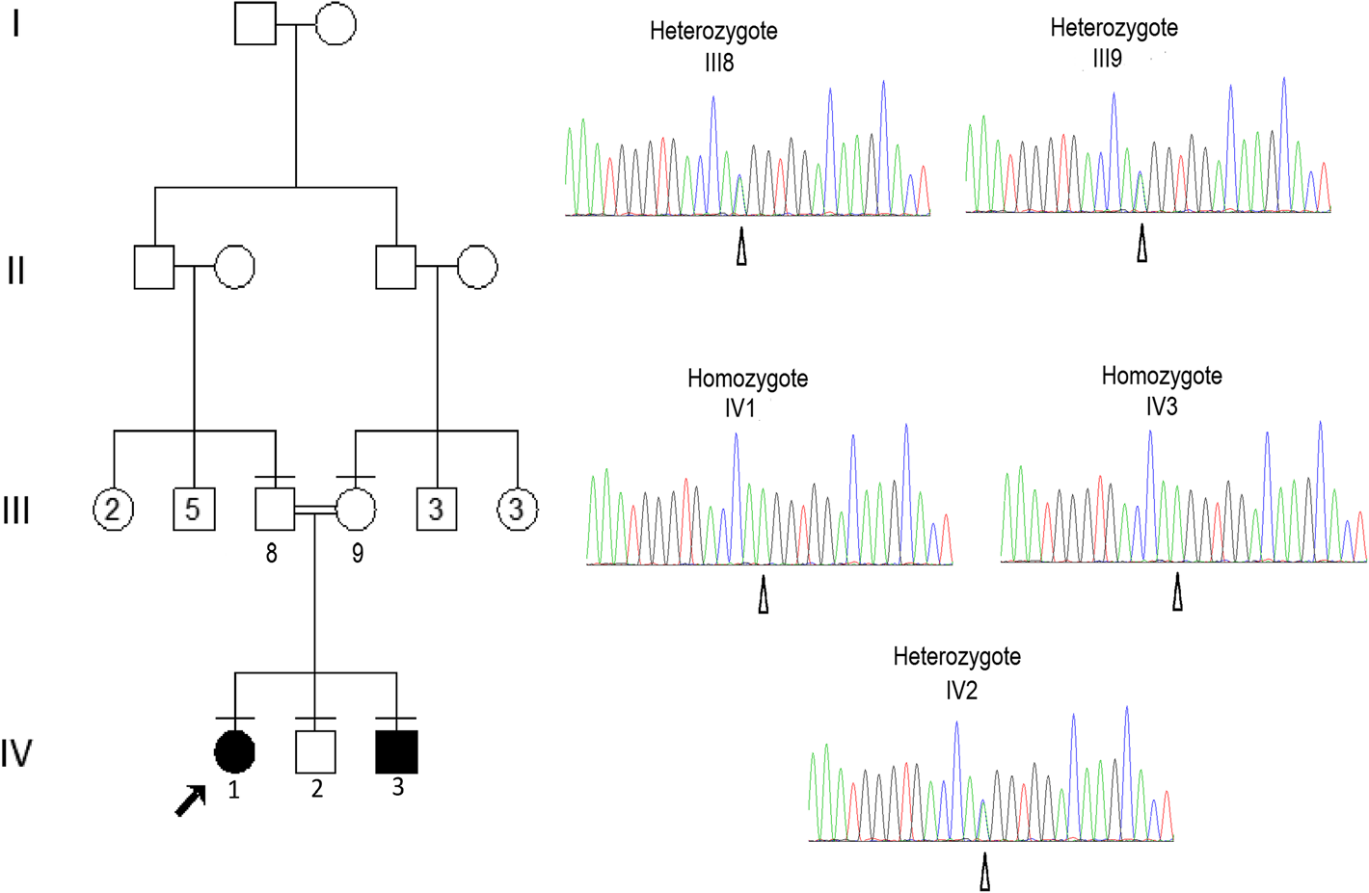
Family pedigree. The proband is noted by an *arrow*. Family members for which DNA was available for testing are noted by a *horizontal line* above the individual’s symbol. Proband’s DNA was used for exome sequencing. Sanger sequencing chromatograms show that patients are homozygous while the parents and non-affected sibling are heterozygous

**Fig. 2:**
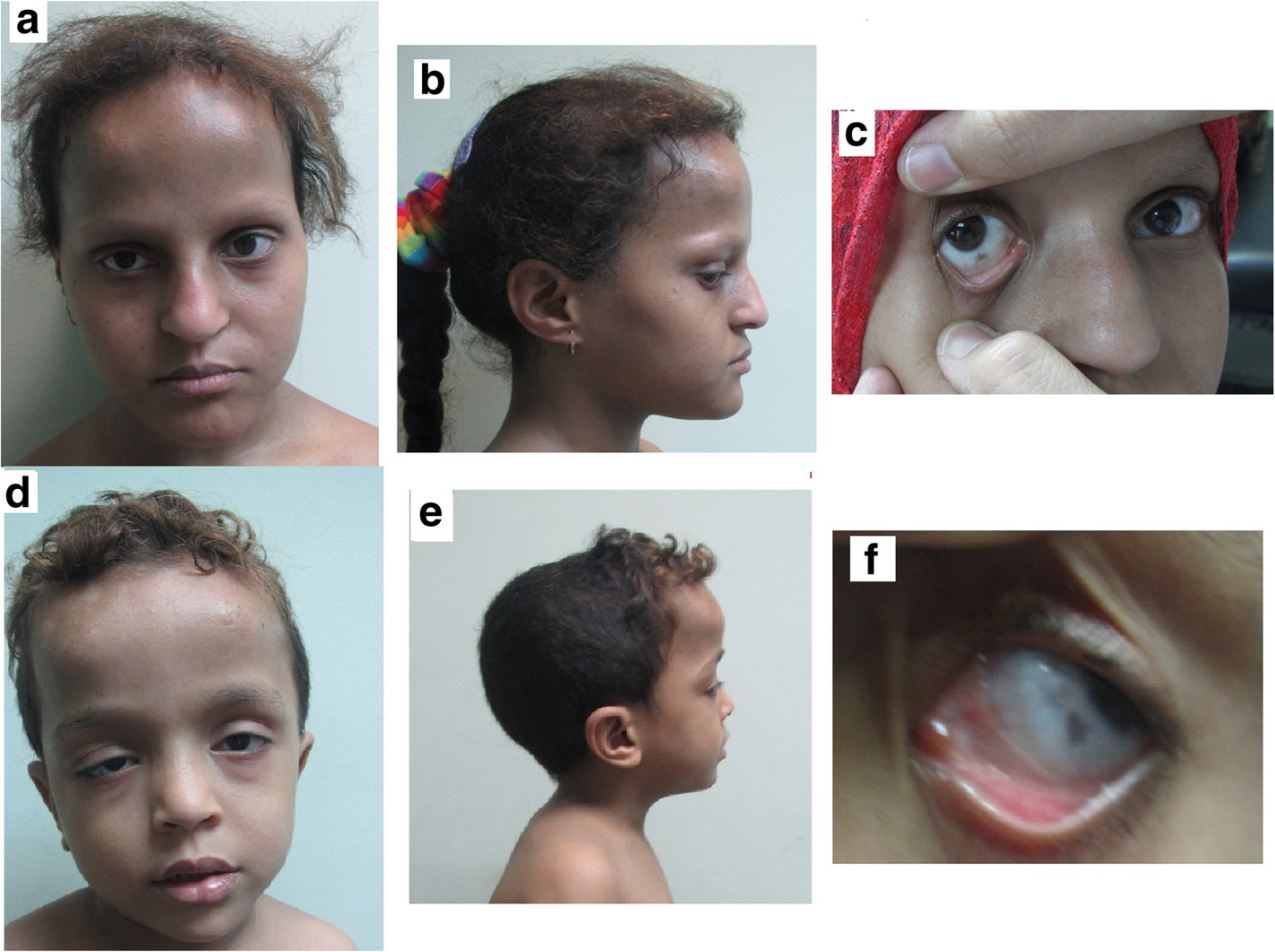
Phenotypes of proband IV1 and brother IV3. **a**, **b** Proband’s facial features with high forehead, prominent nose, and sparse eyebrows and eyelashes. **c** Scleral pigmentation in proband. **d**, **e** Facial features with high forehead, prominent nose sparse eyebrows and eyelashes, and unilateral ptosis in brother IV3. **f** Scleral pigmentation in brother IV3

Figure 1 shows the pedigree of the consanguineous family originating from Egypt. The parents of two affected offspring are paternal first cousins. The eldest affected is a girl (IV:1), first seen at the genetic clinic at the age of 13 years with intellectual disability, short stature, and delayed puberty. Her height was 128 cm (−5.1SD), weight was 25 kg (−2.3SD), and head circumference (HC) was 51 cm (−2SD). Birth history was uncomplicated. She had hypotonia and motor delay; sat unsupported at 2 years and walked at 3 years. Speech development was also delayed. At 13 years of age, she shows hypotonia and intellectual disability (IQ = 58, Stanford-Binet scale). Her dysmorphic facial features (Fig. 2) include sparse eyebrows and eyelashes, pigmented sclerae, high forehead, prominent nose, low-set ears, and abnormal palmar creases. At 13 years, she showed no signs of puberty with axillary hair at A1, pubic hair at P1, breast development at B1, and absence of menarche. Investigations revealed a deficiency of growth hormone as measured by ITT and clonidinetesting. Radiologic examination revealed delayed bone age. Brain MRI, EEG, hearing and ophthalmologic testing were without abnormalities. Banded karyotype was 46, XX. The proband was reexamined at the age of 15 6/12 years showing a height of 135 cm (−3.8SD), weight of 32 kg (−2.3SD), and HC of 51.2 cm (−2.2 SD). Menarche was reached at the age of 15 years.

The affected brother (IV:3) (Fig. 1) was first seen at the genetic clinic at the age of 5 years. He was delivered by cesarean section and noticed to have unilateral ptosis at birth. He had hypotonia; sitting unsupported at 2 years and walking at 3.5 years. Speech development was also delayed with first words at 2.5 years. Examination revealed a height of 85 cm (−4.8SD), weight of 13 kg (−3SD), and HC of 50 cm (−1.2SD). He has intellectual disability with similar facies to his affected sister (Fig. 2) including sparse eyebrows and eyelashes, pigmented sclerae, unilateral ptosis, high forehead, prominent nose, long philtrum, low-set ears, and abnormal palmar creases. Investigations have shown similar results to his sister with growth hormone deficiency, delayed bone age, and no abnormalities in brain MRI, EEG, hearing, and ophthalmologic testing. Karyotype was 46, XY. He was reexamined at the age of 7 years showing a height of 94 cm (−4.8SD), weight of 14 kg (−2.9SD) and HC of 52 cm (within the mean) with intellectual disability (IQ = 68, Stanford-Binet scale).

#### Genetic analysis

**Fig. 3:**
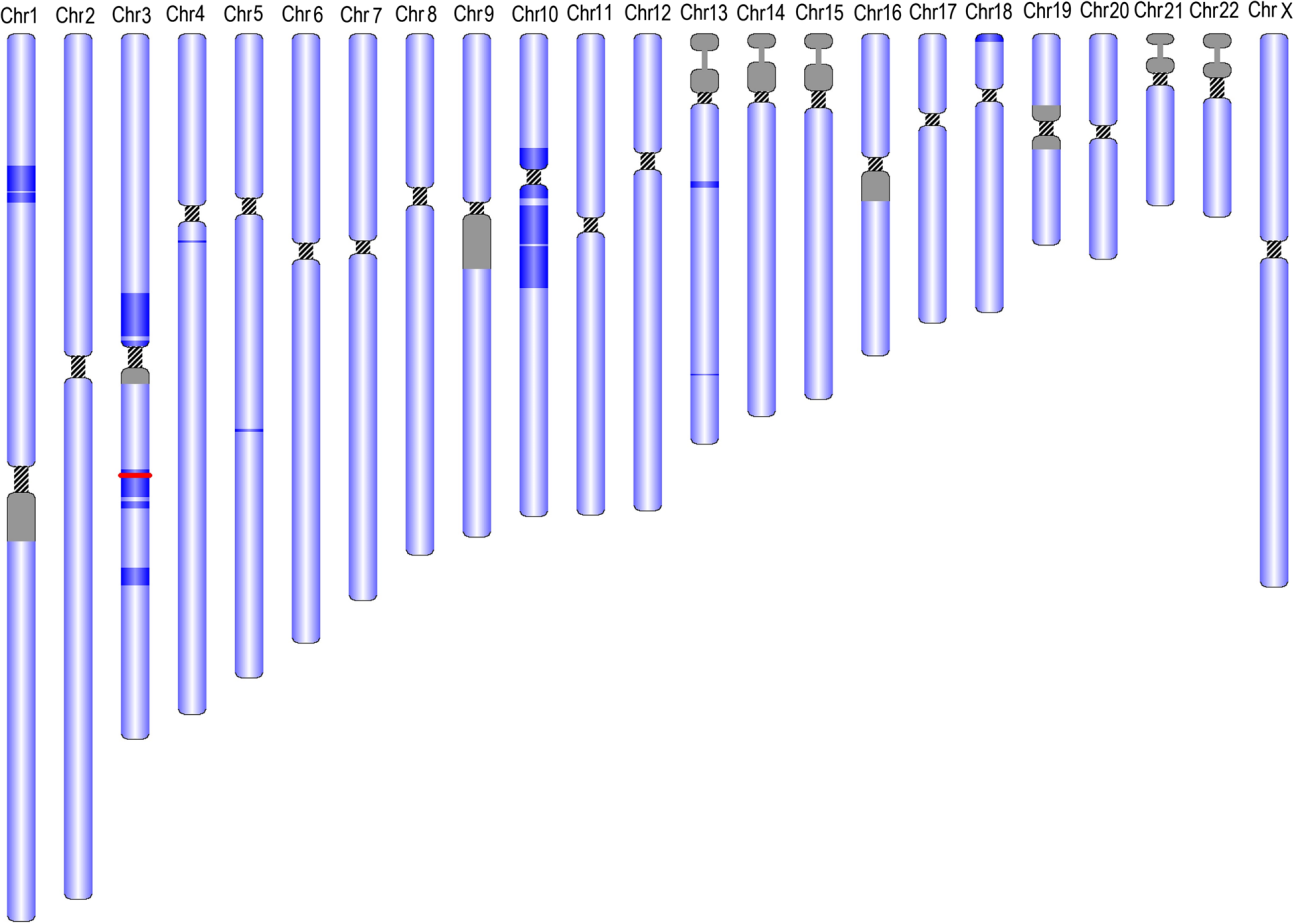
ROH and identification of KALRN. The *dark blue areas* demarcate the positions of the genome that are homozygous and respect the familial segregation. The *horizontal red line* shows the position of KALRN in chromosome 3. *Gray areas* designate the small arms of the acrocentric chromosomes and known heterochromatic areas (figure made through http://db.systemsbiology.net/gestalt/cgi-pub/genomeMapBlocks.pl)

In both parents, 56 ROH were detected with a total size of 44 and 45.8 Mb and average size of 785 and 817 kb, respectively. In the children, the equivalent values were 58, 65, and 70 ROH, with a total size of 365, 312, and 114 Mb and average size of 6.3, 4.8, and 1.6 Mb, respectively. The combination of the ROH provided the position of the target areas in which the candidate genes would be searched (Fig. 3).

After exome sequencing, 139,353,457 unique reads were on target, resulting in coverage of at least eight times for 98.1 % of the protein coding fraction of the genome (RefSeq, version 58). Twenty-two thousend seven hundred sixty exonic variants of high quality were detected, 11,373 of which were synonymous (50.1 %), 10,044 (44.25 %) were missense, 74 (0.33 %) were nonsense, and 653 were indels (2.87 %).

**Fig. 4:**
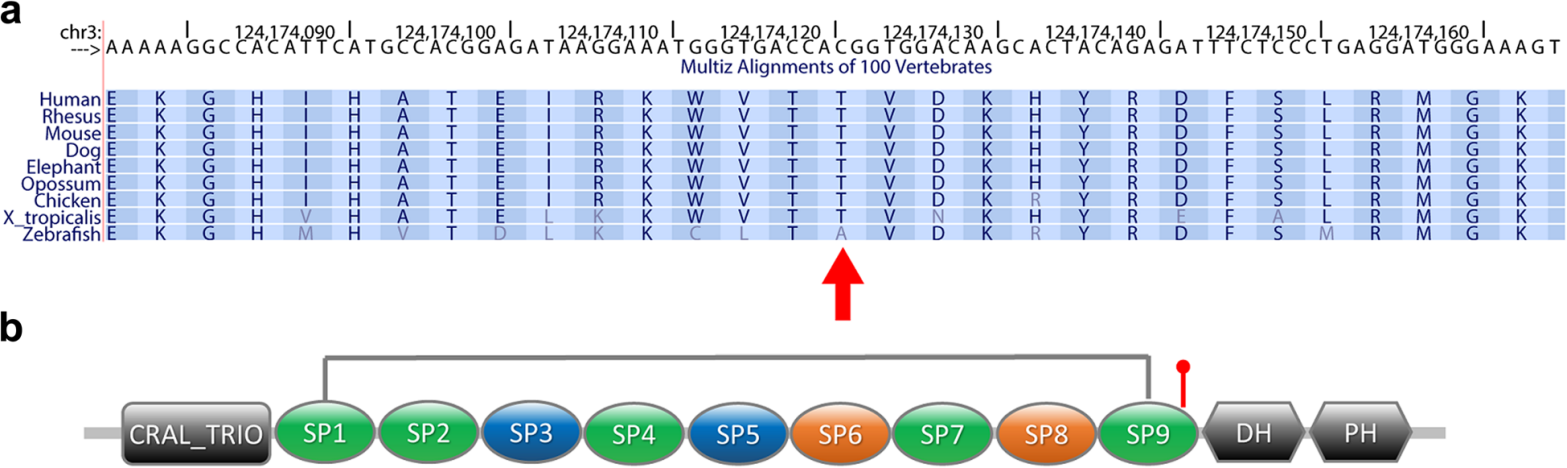
Conservation and position on the protein of the changed amino acid. **a** The *red arrow* shows the changed bp and the corresponding amino acid. NC_000003.11:g.124174121C>A (hg19), NM_003947.4:c.3644C>A:p.(Thr1215Lys). The area is very well conserved throughout the species. **b** Graph of the protein, showing the nine spectrin domains. The position of the changed aminoacid is represented by the *red arrow* (figure adapted from Vishwanatha et al. [26]). *CRAL_TRIO* CRAL-TRIO domain, *SP* spectrin domain, *DH* Dbl homology domain, *PH* Pleckstrin homology domain. SP domains in *green* are identified by Uniprot (http://www.uniprot.org/) and Interpro (http://www.ebi.ac.uk/interpro/). SP domains in *blue* are identified only by Interpro. SP domains in *orange* were identified after manual curation by Vishwanatha et al. [26]

Three variants passed the frequency criterion (MAF <0.01) and respected the segregation in the family. Of these, only one was predicted to be pathogenic by at least two pathogenicity prediction programs, NM_003947.4:c.3644C>A:p.(Thr1215Lys) in *KALRN*. The values predicted by SIFT [23], PolyPhen2 [24], and Mutation Taster [25] were 0.02, 0.8, and 0.996, respectively, and the region is very well conserved throughout the species (Fig. 4a). The variant was found in one of the previously defined target genomic areas of 6.6 Mbp (Fig. 3). Sanger sequencing confirmed the homozygosity in the affected individuals and the heterozygosity of the identified variant in both parents and the non-affected sibling. The variant is found in the ninth and last spectrin repeat of kalirin [26]. The spectrin repeats of kal7 are important for its role in spine morphogenesis [27, 28] (Fig. 4b).

The other two variants which were not predicted to be pathogenic were identified in *CFAP57* (previously named *WDR65*) (NM_001195831.2:c.3299C>T:p.(Pro1100Leu)) and in the last exon in *DPH2* (NM_001384.4:c.1429C>T:p.(Arg477*)). *CFAP57* was previously correlated with a Van der Woode syndrome (VDW) variant [29] but was later reclassified as variant of unknown clinical significance (VUS). Scarce data is known for *DPH2* gene. In mouse, the function of the gene is mostly related to aging and to the skeleton, with expression in liver and biliary tissue. This data led us to consider this variant as unlikely to cause the phenotype in this family [30, 31].

### Discussion

Kalirin is a prime candidate for a role in ID since alterations in signaling pathways involving the Rho family of small GTPases, key regulators of the actin, and microtubule cytoskeletons contribute to both syndromic and non-syndromic intellectual disability disorders [32]. In mice, loss of one or both copies of *Kalrn* leads to reduction in neuron spine densities in certain brain regions [33]. Heterozygote and knockout mice showed variable impairments in cognitive functions related to working memory, social recognition, and social approach, demonstrating the role of kalirin in the regulation of cortical ultrastructure and spine structural plasticity [34]. Elimination of Kalrn expression in POMC cells reduces anxiety-like behavior in mice [35]. Kalirin-7 in rodents (equivalent to human NM_003947.4) is the most abundant kalirin isoform in the adult rodent brain and is localized at the postsynaptic side of excitatory synapses [36, 37]. Knockout and overexpression studies revealed important roles for Kal7 in dendritic spine formation and synaptic function in rodents [16, 33, 34, 36].

The processes that regulate the morphological development of dendrites and dendritic spines have a significant impact on the establishment and function of synapses and on neuronal circuits [16]. The major functional group of ID-related proteins corresponds to proteins enriched at synaptic compartments [38], and investigations in children and adolescents with unclassified ID confirm the reduced density and spine dysgenesis in apical dendrites of the prefrontal cortex [39]. Abnormal neural cell spine morphology was the only anatomical alteration reported in cases of non-syndromic ID [33]. A rare coding variant, kalirin-7-D1338N, was identified in a schizophrenia patient and his sibling with major depressive disorder where both subjects carrying the polymorphism displayed reduced cortical volume in the superior temporal sulcus (STS), a region implicated in schizophrenia [40]. These data suggest that single amino acid changes in proteins involved in dendritic spine function can have significant effects on the structure and function of the cerebral cortex [40]. Other reports have indicated that dysfunctions in Rho-GEF signaling pathways are associated with various ID syndromes [41] and with Alzheimer disease [42]. Moreover, a correlation has been reported between the levels of Kalirin expression and the pathology of dendritic spines in some psychiatric and neurological disorders such as Huntington’s disease, Alzheimer’s disease, ischemic stroke, schizophrenia, depression, and cocaine addiction [43, 44].

In the tested family, there were clinical features other than ID, which could be correlated with Kalirin dysfunction. Both patients suffer from short stature and growth hormone deficiency. Mice with ablated spectrin domains of Kalirin had deficient growth rate and dysfunctional secretion of growth hormone [17]. Hypotonia was also present in our patients and may be related to the pre- and postsynaptic deficits in the neuromuscular junction reported in mice [17].

Kalirin has also been implicated in bone homeostasis where its deletion was reported to affect directly osteoclast and osteoblast activity. It may also play a role in paracrine and/or endocrine signaling events that control skeletal bone remodeling and the maintenance of bone mass [18]. This could be consistent with the observed delayed bone age in the patients.

The other two variants identified in *CFAP57* and in the last exon in *DPH2* were not predicted to be pathogenic. The patients in the studied family do not have any of the features of VWS including the pits and/or sinuses of the lower lip, and cleft lip and/or cleft palate, which leads us to consider *CFAP57* variant as a VUS in respect to the patient’s phenotype. *DPH2*, one of several enzymes involved in the synthesis of diphthamide linked to diphtheria toxin (OMIM 603456), was also considered as unlikely to cause the phenotype in this family.

### Conclusions

In this report, the clinical evaluation combined with the power and efficiency of genomic analysis defined a new candidate gene, *KALRN*, as the possible underlying cause of the syndromic autosomal recessive ID in the studied family. The role of *KALRN* in causing cognitive impairment in human and the full phenotypic spectrum will be established when other families with ID show pathogenic variants in *KALRN* and the exact molecular pathophysiology is understood.

## Abbreviations

ID, intellectual disability; OMIM, Online Mendelian Inheritance in Man (http://omim.org/); ROH, run of homozygosity
